# Analgesic comparison between perineural and intravenous dexamethasone for shoulder arthroscopy: a meta-analysis of randomized controlled trials

**DOI:** 10.1186/s13018-022-02952-6

**Published:** 2022-02-17

**Authors:** Liangku Huang, Peng Li, Liang Zhang, Guangming Kang, Haizhen Zhou, Zandong Zhao

**Affiliations:** 1 Department of Hand Surgery, Xi’an Honghui Hospital, Xi’an Jiaotong University Health Science Center, Xi’an, 710054 Shaanxi China; 2 Department of Orthopaedic Oncology, Xi’an Honghui Hospital, Xi’an Jiaotong University Health Science Center, Xi’an, 710054 Shaanxi China; 3 Sports Medicine Center, Xi’an Honghui Hospital, Xi’an Jiaotong University Health Science Center, No. 555 Youyidong Street, Beilin District, Xi’an, 710054 Shaanxi China

**Keywords:** Perineural dexamethasone, Intravenous dexamethasone, Shoulder arthroscopy, Pain management, Randomized controlled trials

## Abstract

**Introduction:**

The analgesic comparison between perineural and intravenous dexamethasone on interscalene block for pain management after shoulder arthroscopy remains controversial. We conduct this meta-analysis to explore the influence of perineural versus intravenous dexamethasone on interscalene block for pain control after shoulder arthroscopy.

**Methods:**

We have searched PubMed, Embase, Web of science, EBSCO and Cochrane library databases through April 2021 and included randomized controlled trials (RCTs) assessing the effect of perineural and intravenous dexamethasone on interscalene block in patients with shoulder arthroscopy.

**Results:**

Five RCTs were included in the meta-analysis. Overall, compared with intravenous dexamethasone for shoulder arthroscopy, perineural dexamethasone led to similar block duration (SMD = 0.12; 95% CI − 0.12 to 0.35; *P* = 0.33), pain scores at 12 h (SMD = − 0.67; 95% CI − 1.48 to 0.15; *P* = 0.11), pain scores at 24 h (SMD = − 0.33; 95% CI − 0.79 to 0.14; *P* = 0.17), opioid consumption (SMD = 0.01; 95% CI − 0.18 to 0.19; *P* = 0.95) and incidence of nausea/vomiting (OR = 0.74; 95% CI 0.38–1.44; *P* = 0.38).

**Conclusions:**

Perineural and intravenous dexamethasone demonstrated comparable pain relief after shoulder arthroscopy.

## Introduction

Arthroscopy has been widely accepted to diagnose and treat shoulder diseases [1–3]. However, significant postoperative pain is the main concern after this surgery and effective analgesia is required for this day-case surgery [3–5]. Interscalene brachial plexus block (ISB) is the standard analgesia after shoulder surgery with the features of superior analgesia and reduced opioid consumption [6–8]. ISB is limited by short analgesic maintenance for several hours, and especially moderate to severe pain of this surgery requires opioid supplementation [9].

The increase in the dose of local anesthetic is used to prolong ISB, but has the limitation of narrow therapeutic window and volume/concentration. Volumes of 10 ml or greater injected into the interscalene groove can increase the risk of ipsilateral hemi-diaphragmatic paresis [10]. Several anesthetics have been developed to prolong ISB. In particular, dexamethasone used by perineural approach showed the potential in prolonging the duration of peripheral nerve blocks when in conjunction with local anesthetics [11].

Recently, several studies have compared the analgesic efficacy between perineural with intravenous dexamethasone for the pain management after shoulder arthroscopy, but the results are conflicting [10, 12, 13]. With accumulating evidence, we therefore perform this meta-analysis of RCTs to compare perineural with intravenous dexamethasone for shoulder arthroscopy.

## Materials and methods

Ethical approval and patient consent were not required because this was a meta-analysis of previously published studies. We conducted this meta-analysis in adherence to PRISMA (Preferred Reporting Items for Systematic Reviews and Meta-Analyses) [14, 15].

### Search strategy and study selection

Two investigators have independently searched the following databases (inception to April 2021): PubMed, Embase, Web of science, EBSCO and Cochrane library databases. The electronic search strategy was conducted using the following keywords: “dexamethasone” AND “interscalene block” AND “shoulder arthroscopy.” We also checked the reference lists of the screened full-text studies to identify other potentially eligible trials.

The inclusive selection criteria were as follows: (i) patients underwent shoulder arthroscopy; (ii) intervention treatments were perineural versus intravenous dexamethasone as the adjunctive therapy to interscalene block; (iii) study design was RCT.

### Data extraction and outcome measures

We extracted the following information: author, number of patients, age, female, body weight, American Society of Anesthesiologists (ASA) physical status and detail methods in each group etc. Data were extracted independently by two investigators, and discrepancies were resolved by consensus. We also contacted the corresponding author to obtain the data when necessary. The primary outcome was block duration. Secondary outcomes included pain scores at 12 h, pain scores at 24 h, opioid consumption, and the incidence of nausea/vomiting.

### Quality assessment in individual studies

Methodological quality of the included studies was independently evaluated using the modified Jadad scale [16]. There were three items for Jadad scale: randomization (0–2 points), blinding (0–2 points) and dropouts and withdrawals (0–1 points). The score of Jadad Scale varied from 0 to 5 points. An article with Jadad score ≤ 2 was considered to be of low quality, while Jadad score ≥ 3 suggested high quality [17].

### Statistical analysis

We estimated the standard mean difference (SMD) with 95% confidence interval (CI) for continuous outcomes (block duration, pain scores at 12 h, pain scores at 24 h and opioid consumption) and odd ratios (ORs) with 95% CIs for dichotomous outcomes (nausea/vomiting). The random-effects model was used regardless of heterogeneity. Heterogeneity was reported using the I^2^ statistic, and I^2^ > 50% indicated significant heterogeneity [15, 18]. We searched for potential sources of heterogeneity via omitting one study in turn for the meta-analysis or performing subgroup analysis. All statistical analyses were performed using Review Manager version 5.3 (The Cochrane Collaboration, Software Update, Oxford, UK).

## Results

### Literature search, study characteristics and quality assessment

**Fig. 1:**
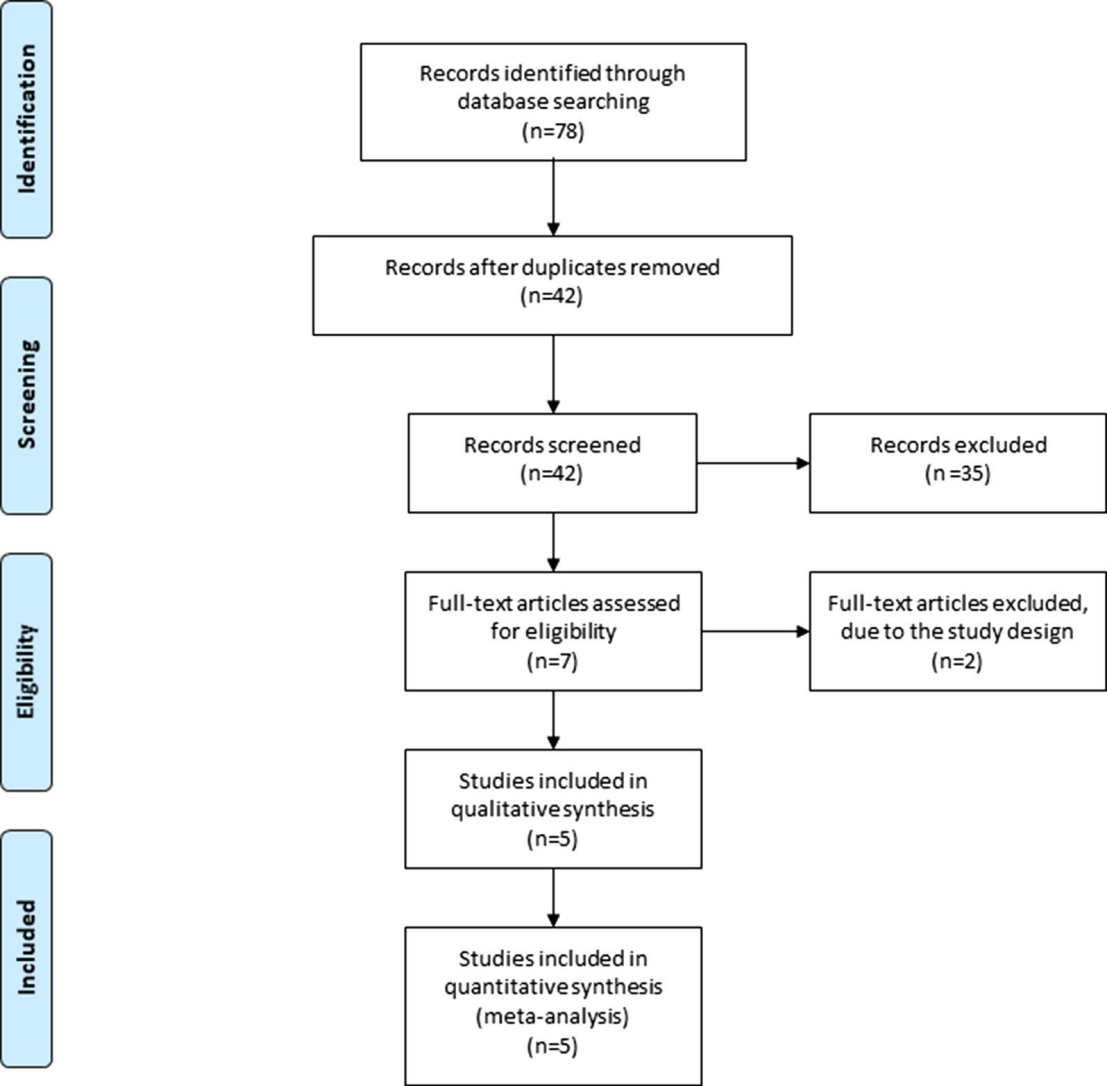
Flow diagram of study searching and selection process

Figure 1 demonstrates the detailed flowchart of the search and selection results. Initially, 78 potentially relevant articles were identified and five RCTs were finally included in the meta-analysis [10, 12, 13, 19, 20]. The baseline characteristics of five eligible RCTs in the meta-analysis are summarized in Table 1. The five studies were published between 2016 and 2020, and total sample size was 585.

**Table 1:** Characteristics of included studies

References	Perineural dexamethasone group						Intravenous dexamethasone group						Jada scores
	Number	Age (years)	Female (n)	Weight (kg)	ASA physical status (I/II/III)	Methods	Number	Age (years)	Female (n)	Weight (kg)	ASA physical status (I/II/III)	Methods	
McHardy et al. [10]	92	51.6 (18–73)	25	–	25/49/18	Interscalene block analgesia supplemented with perineural dexamethasone 4 mg	90	52.8 (22–76)	21	–	18/57/15	Interscalene block analgesia supplemented with intravenous dexamethasone 4 mg	5
Kahn et al. [12]	63	50 ± 14	26	–	22/38/3	Interscalene block supplemented with perineural dexamethasone 1 mg	62	47 ± 15	23	–	26/33/3	Interscalene block analgesia supplemented with intravenous dexamethasone 1 mg	5
Holland et al. [13]	70	54 ± 12	21	87 ± 16	24/40/6	Interscalene block analgesia supplemented with perineural dexamethasone 4 mg	69	53 ± 14	16	89 ± 17	21/44/5	Interscalene block analgesia supplemented with intravenous dexamethasone 4 mg	4
Sakae et al. [19]	20	53.2 ± 9.8	8	63.2 ± 5.1	9/11/0	Interscalene block analgesia supplemented with perineural dexamethasone 4 mg	20	52.1 ± 12.3	6	65.3 ± 4.2	8/12/0	Interscalene block analgesia supplemented with intravenous dexamethasone 4 mg	3
Chun et al. [20]	50	50.8 ± 17.5	17	69.6 ± 12.9	24/26/0	Interscalene block analgesia supplemented with perineural dexamethasone 5 mg	49	53.0 ± 14.2	15	68.0 ± 11.6	17/32/0	Interscalene block analgesia supplemented with intravenous dexamethasone 5 mg	4

Data were presented as mean ± SD or median (IQR)

The doses of perineural or intravenous dexamethasone ranged from 1 to 5 mg, and the concentrations of perineural dexamethasone varied from 0.1333 mg/ml to 1 mg/ml. Among the five studies included here, three studies reported block duration [10, 12, 13], three studies reported pain scores at 12 h [10, 19, 20], four studies reported pain scores at 24 h [10, 12, 19, 20], three studies reported opioid consumption [10, 12, 13], and three studies reported nausea/vomiting [10, 19, 20]. Jadad scores of the five included studies varied from 3 to 5, and all five studies had high quality according to quality assessment.

### Primary outcome: block duration

**Fig. 2:**
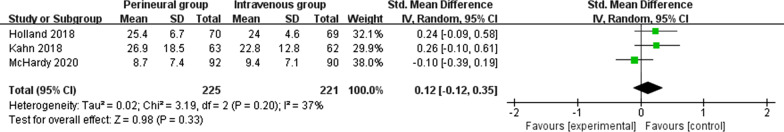
Forest plot for the meta-analysis of block duration

These outcome data were analyzed with the random-effects model, and compared to intravenous dexamethasone for shoulder arthroscopy, perineural dexamethasone resulted in comparable duration of sufficient analgesia, as evidenced by similar block duration (SMD = 0.12; 95% CI − 0.12 to 0.35; *P* = 0.33) with low heterogeneity among the studies (I^2^ = 37%, heterogeneity *P* = 0.33) (Fig. 2).

### Sensitivity analysis

Low heterogeneity was observed among the included studies for the primary outcome, so we did not perform sensitivity analysis via omitting one study in turn to detect the heterogeneity.

### Secondary outcomes

**Fig. 3:**
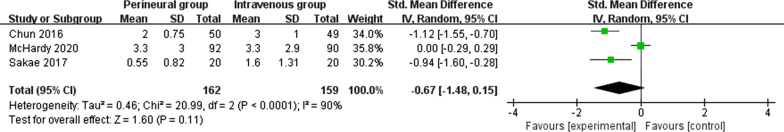
Forest plot for the meta-analysis of pain scores at 12 h

**Fig. 4:**
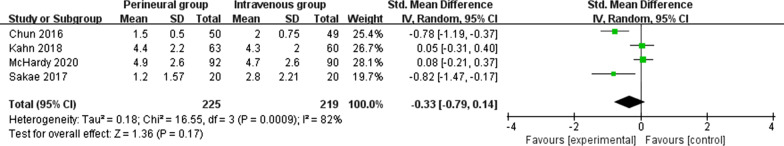
Forest plot for the meta-analysis of pain scores at 24 h

**Fig. 5:**
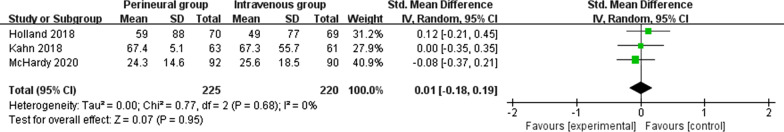
Forest plot for the meta-analysis of opioid consumption

**Fig. 6:**
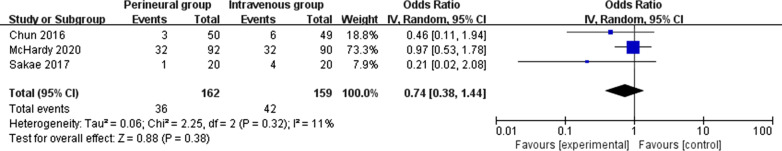
Forest plot for the meta-analysis of nausea/vomiting

In comparison with intravenous dexamethasone for shoulder arthroscopy, perineural dexamethasone exhibited comparable control of pain intensity shown by pain scores at 12 h (SMD = − 0.67; 95% CI − 1.48 to 0.15; *P* = 0.11; Fig. 3) and 24 h (SMD = − 0.33; 95% CI − 0.79 to 0.14; *P* = 0.17; Fig. 4). In addition, these two approaches of dexamethasone resulted in similar opioid consumption (SMD = 0.01; 95% CI − 0.18 to 0.19; *P* = 0.95; Fig. 5) and the incidence of nausea/vomiting (OR = 0.74; 95% CI 0.38–c1.44; *P* = 0.38; Fig. 6).

## Discussion

Serious pain after shoulder arthroscopy commonly occurs and mainly results from the insertion of arthroscopic instruments into the joint, soft tissue dissection and distention [21–25]. Patients’ early mobilization and rehabilitation is significantly affected by this postoperative pain [26–28]. Numerous techniques have been studied, and ISB is widely accepted as the most effective analgesic technique for this surgery [3, 29–31]. Furthermore, supplementation with dexamethasone revealed a significant role in increasing the duration and analgesic efficacy of ISB for shoulder arthroscopy [13, 19].

Previous study comparing perineural and systemic dexamethasone showed that both routes were associated with prolonged and similar block duration [32–34]. In order to compare perineural with intravenous dexamethasone supplementation for ISB in patients with shoulder arthroscopy, our meta-analysis included five RCTs and revealed that perineural and intravenous dexamethasone resulted in comparable block duration, pain control and opioid consumption when in conjunction with local analgesics for shoulder arthroscopy. Dexamethasone is found to reduce ectopic neuronal discharge and inhibit potassium channel-mediated discharge of nociceptive C-fibers. Additionally, dexamethasone supplementation can provide superior analgesia in the context of peripheral nerve block through systemic anti-inflammatory effects [10, 35].

As shown in Fig. 3, considerable clinical heterogeneity is observed, and we searched for potential sources of heterogeneity via omitting one study in turn. After excluding the study conducted by McHardy et al. [10], we found that no heterogeneity remained and perineural dexamethasone resulted in lower pain scores at 12 h than intravenous dexamethasone (SMD = − 1.07; 95% CI − 1.43 to − 0.71; P < 0.00001). McHardy et al. reported the perineural dexamethasone at the concentration of 0.667 mg/ml [10], while other two studies reported the perineural dexamethasone at the concentration of 0.190 and 0.417 mg/ml [19, 20]. In addition, in Fig. 4, Kahn et al. reported perineural dexamethasone at the concentration of 1 mg/ml [12], and perineural dexamethasone at the concentration of 1 mg/ml and 0.667 mg/ml can obtain the comparable analgesic efficacy than intravenous dexamethasone. These indicated that the lower concentration of perineural dexamethasone (≤ 0.417 mg/ml) produced substantially lower analgesic efficacy than intravenous dexamethasone for shoulder arthroscopy, and higher concentration of perineural dexamethasone (≥ 0.667 mg/ml) and intravenous dexamethasone had comparable analgesic efficacy, suggesting that concentrations of perineural dexamethasone were crucial for the analgesic efficacy of interscalene block in patients with shoulder arthroscopy.

In addition, the incidence of nausea/vomiting was similar between two groups based on our results. This meta-analysis also has several limitations. Firstly, our analysis is based on five RCTs, and two of them have a relatively small sample size (n < 100). Overestimation of the treatment effect is more likely in smaller trials compared with larger samples. Next, different concentrations and combination methods of dexamethasone may produce some bias. Finally, it is not feasible to perform the meta-analysis of some important index such as discharge time and time to first analgesic requirement based on current RCTs.

## Conclusions

Perineural and intravenous dexamethasone showed similar efficacy for block duration after shoulder arthroscopy.

## Data Availability

Not applicable.

## Abbreviations

RCTs: Randomized controlled trials
MDs: Mean differences
CIs: Confidence intervals
RRs: Risk ratios
